# Unanticipated Changes in Drug Overdose Death Rates in Canada During the Opioid Crisis

**DOI:** 10.1007/s11469-022-00932-9

**Published:** 2022-10-10

**Authors:** John Snowdon, Namkee Choi

**Affiliations:** 1 Discipline of Psychiatry, Sydney Medical School, Sydney, NSW Australia; 2 Centre for Mental Health, Concord Hospital, Concord, NSW 2139 Australia; 3 Steve Hicks School of Social Work, University of Texas at Austin, Austin, TX USA

**Keywords:** Suicide, Canada, Accidental poisoning, Overdose deaths, Opioid crisis, Mortality rates

## Abstract

Escalating drug overdose death rates in Canada are of ever-increasing concern. To better understand the extent of this health threat, we obtained mortality statistics and population figures for the years 2000 to 2020, and examined rates of overdose deaths, coded (using ICD-10) as accidental, suicide or “undetermined intent.” The drug deemed as primarily responsible for the death was categorized as opioid, non-opioid, or unspecified. Age patterns of drug deaths were graphed. Joinpoint analysis was used to test the significance of changes in death rates. Accidental opioid and stimulant overdose death rates in Canada have climbed faster since 2011, though not as high as corresponding US rates. Unknown cause death rates have increased. However, opioid and non-opioid suicide rates have decreased significantly since 2011, and there have been fewer drug deaths of undetermined intent. Increased attention to the possibility that some suicides are being misclassified is warranted.

Widespread use of prescription opioids and a dramatic increase in the availability of illicit opioids throughout North America in the 2000s have resulted in a so-called opioid epidemic (Skolnick, 2018). The extent to which the drug crisis has affected North America is unmatched by other regions across the globe. The epidemic is associated with reduced life expectancy in the USA and Canada (Kerr, 2019). Opioid overdoses and deaths in Canada have led to a “deadly and ongoing public health crisis” (Government of Canada, 2022).

Member States of the World Health Organization (WHO) use the International Classification of Diseases (ICD-10; WHO, 1992) when reporting cause-of-death (c.o.d.) statistics for inclusion in the WHO Mortality Database. Drug-induced deaths are classified according to their intent—accidental, intentional (assault or suicide), or event of undetermined intent (EUI). Presenting rates per 100,000 persons per year, between 2000 and 2011, the crude rate of *all* drug overdose deaths rose from 4.74 to 7.41, the rate of opioid-related deaths in Canada having increased relatively slowly, from 1.71 to 2.21 (Skinner et al., 2016). The rate of deaths coded as drug suicides rose during this period, from 1.63 to 2.14.

Between 2011 and 2017, the opioid-related death rate in Canada increased further, with Alberta and British Columbia (BC) showing higher rates than other provinces. Of 2946 deaths in 2016, 88% were reportedly accidental, 8.5% suicides, and 3.5% EUI (Government of Canada, 2018).

From 2015, there was a steep rise in fentanyl-related deaths in Canada. The highest percentage of such deaths occurred among males aged 30–39 years (GoC, 2018; Belzak & Halverson, 2018). The opioid-related death rate rose to 20.0 during 2021 (GoC, 2022) following emergence and then worsening of the COVID-19 pandemic, and the adoption of preventive measures. Fentanyl was involved in 86% of all accidental opioid toxicity deaths in 2021 (GoC, 2022). By comparison, the drug overdose death rate in the USA in 2020 was provisionally calculated as 28.3; almost 75% involved opioids, the rate being 21.6 (Centers for Disease Control and Prevention, 2022). Of these, 82% involved synthetic opioids and 18% involved prescription opioids.

Along with increasing opioid use in North America in the early 2000s, psychostimulant use also increased (Belzak & Halverson, 2018; Fischer et al., 2021). Methamphetamine was identified as a main risk for severe mental health problems (including suicide), especially among high-risk drug-using populations. Data show that 59% of apparently accidental opioid toxicity deaths in 2021 also involved a stimulant and that 86% of apparently accidental *stimulant* toxicity deaths in 2021 also involved an opioid (GoC, 2022), reflecting the polysubstance nature of the crisis. Of these stimulant deaths, 71% involved cocaine, 46% methamphetamines. A majority were of persons aged 20 to 49 years.

## Drug Suicides and EUI Deaths

The overall age-standardized suicide rate in Canada decreased between 2000 and 2011, from 14.4 to 12.6, with the gas poisoning suicide rate falling from 1.17 to 0.61 (Skinner et al., 2016). There was, however, an increase in the rates of drug suicide (1.63 to 2.14) and EUI drug deaths (0.53 to 1.28). In Alberta (the Canadian province with the second highest rate of opioid-related deaths), in 2000–2016, drug overdoses accounted for 20% of suicides, and of these, 22% involved opioids; the average rate of opioid suicide was 0.61 for females and 0.57 for males (Chan et al., 2018). By comparison, the US opioid suicide rate rose from 0.27 to 0.58 between 1999 and 2017, but the percentage of opioid-related deaths that were suicides fell from 9 to 4% (Olfson et al., 2019). Unlike in Canada, where total suicide rates fell during 2011–2018, US reports showed continuously increasing annual rates of *total* suicides during 1999–2018, from 10.5 to 14.8 (CDC, 2022).

Given the paucity of recent research on drug-related suicide rates at the national level, we examined Canada’s mortality data to assess whether, along with changes in death rates attributed to opioid and/or stimulant overdose, there have been corresponding changes in rates of drug-related suicides and EUI deaths. A positive association between rates of accidental opioid overdose deaths and drug suicides over the last two decades could be expected for the following reasons: (1) substance misuse is a known risk factor for suicide (Bohnert et al., 2017; Rontziokos & Deane, 2019); (2) Canadian doctors in the early 2000s were being persuaded to increase their prescribing and dosage of opioids, for chronic pain, thus making lethal drugs more available to people who already had an increased tendency to suicide. Prescription opioids are known to increase the risk of depression (Scherrer et al., 2014); and (3) availability and ease of access to a lethal means of suicide (opioids, prescribed or illicit) increased.

There is also good reason to expect a finding that rates of EUI deaths increased (at least proportionally) during the years when drug death numbers were escalating. In many cases, following such deaths, no evidence was provided to those certifying cause of death (c.o.d.) about whether or not the decedents intended to die when they overdosed (GoC, 2021). Intent in such cases may fall along a continuum between purely intentional and unintentional (Rockett et al., 2015).

### Study Aims

In the present study using Canadian drug overdose statistics, we first examined data from the years 2000 to 2020, showing rates of drug overdose deaths deemed to be accidental, intentional, or due to EUI, where the drug primarily responsible for the death was either an opioid/narcotic or a drug from another category, or where no one type of drug had been specified as primarily responsible for the death. Next, we examined whether there were significant sex and age group differences between rates of accidental overdose deaths and of drug suicides. Third, we explored recent changes over time in rates of deaths involving a combination of drug types, most commonly opioids with stimulants. Fourth, in recognition that suicide is widely believed to be under-reported around the world and that inaccuracies have been largely attributed to misclassification of c.o.d. as accident, EUI, or “ill-defined and unknown cause of death” (ICD-10 code R99) (Snowdon & Choi, 2020), we examined data on the numbers of deaths in Canada coded R99 in the last two decades. We hypothesized that increasing drug overdose rates in Canada in the last decade would be associated with increased rates of drug deaths coded as suicide or as EUI. This descriptive study was intended to provoke discussion and questions concerning recent changes in opioid and other drug overdose mortality rates in Canada.

## Methods

### Data Sources

We used online data published by Statistics Canada (2022) regarding numbers of deaths and c.o.d. in Canada each year from 2000 to 2020. Canada has been assessed as providing very high quality c.o.d. data (Mikkelsen et al., 2015). Data were available separately for males and females, and for age groups from birth onwards in gradations of 5 years from 10–14 to 80–84 years, and those aged 85 + years. Population data (males and females in 5-year age groups) for each year were available from Population Pyramid (2021), and data accuracy was ascertained by examining figures from census data recorded in mid-2006, mid-2011, mid-2016, and mid-2021.

### Measures

The following ICD-10 categories used for coding c.o.d. were selected for examination: X60–84 for suicide total; X60–64 for suicide by drug poisoning; X40–44 for accidental death attributed to drugs/medications; and Y10–14 for drug-related EUI deaths. Less than 0.1% of all drug deaths over the 21 years were homicides; thus, they were excluded from this study. Proportionally very few deaths are coded R96 (“other sudden death, cause unknown”) or R98 (“unattended death”), whereas other (i.e., almost all non-infant) “ill-defined and unknown cause deaths” are assigned the ICD-10 code R99; numbers and rates of the latter were also examined.

ICD-10 codes for all accidental, suicide, or EUI drug poisoning deaths indicate whether the underlying c.o.d. was attributable to the following:

- An *opioid/narcotic* (including cocaine) or hallucinogenic [X42, X62, Y12].
- A drug belonging to one of the following *non-opioid* categories and not taken in combination with an opioid: analgesic, anti-pyretic, anti-rheumatic (X40, X60, Y10); psychotropic (including antidepressants, anxiolytics, neuroleptics, psychostimulants), sedative-hypnotic, anti-epileptic, and anti-parkinsonian [X41, X61, Y11]; or acting on the autonomic nervous system [X43; X63; Y13].
- A drug taken in combination with one or more drugs (with an ICD-10 X or Y code different to its own) that may have contributed to the death, but it is uncertain (*unspecified*) which drug was chiefly responsible, or it is an unspecified/unidentified drug, or “other” drug that does not belong to one of the above categories [X44, X64, Y14]. Commonly, stimulants have been assigned to this code, though if not given in combination with other drugs, they should be coded as non-opioid (X41, X61 or Y11).

The names of drugs (e.g., heroin), whether used alone or with other drugs, may also have been coded using so-called “contributing” codes (T codes), but commonly were not. There was no T-code for fentanyl until 2021.

### Analysis

Numbers of deaths per year in Canada, assigned to ICD-10 underlying c.o.d. codes, were totaled. Using population data (males and females separately), crude mortality statistics were calculated for all the groupings listed above. In addition, age patterns of accidental, suicide, and EUI death rates in Canada in 5-year periods, each with a census year as the middle year, were examined and graphed. Numbers of deaths in each male and female 5-year age group, during three 5-year periods, were totaled and mean annual numbers of deaths were noted. Crude mortality statistics were calculated for all age groups by dividing the mean annual number of deaths in the age group under consideration, by the number of persons in that age group during the relevant census year, divided by 100,000. Only the 2014–2018 graphs are shown in this paper. Concerns about high “undetermined cause” mortality rates among deaths in 2019 and 2020 (some being because c.o.d. verdicts had not yet been finalized) led to exclusion of data from those years when reporting on age patterns. Finally, we carried out joinpoint analysis of death rates (Join Point Regression Program, 2020) and tested the significance of annual percentage changes (APC) before and after joinpoints. Statistical significance was set at *p* < 0.05.

## Results

### Drug Overdose Deaths, 2000–2020: Accidental, Suicide, and EUI

**Fig. 1:**
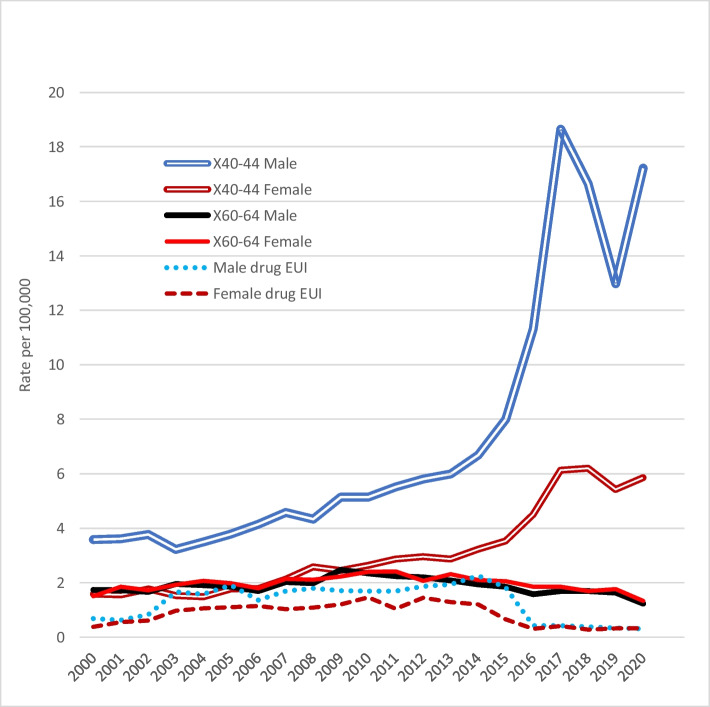
Comparison of male and female accidental versus suicide versus EUI drug overdose death rates. Note: ICD-10 codes: X40–44, accidental poisoning by drugs or medications; X60–64, intentional poisoning by drugs or medications; Y10–14, Deaths attributed to events of undetermined intent (EUI)

The Canadian drug overdose death rate (accidental + intentional + EUI) reached a peak of 14.6 (male 20.8, female 8.4) in 2017; in 2020, Statistics Canada figures showed it as 13.1. Figure 1 shows changes in male and female rates of accidental drug overdose deaths between 2000 and 2020, and compares these with corresponding male and female drug overdose deaths certified as suicides, and with those registered as EUI drug deaths. The trebling of the male accidental drug mortality rate and doubling of the corresponding female rate, between 2013 and 2017, contrast with a slow but progressive fall in the male drug suicide rate from 2009 onwards, and of the female rate somewhat later. The male accidental drug overdose death rate decreased in 2018 and even more so in 2019, but the rate markedly increased again in 2020. The female rate increase slowed in 2018, fell a little in 2019, and rose a little in 2020.

**Fig. 2:**
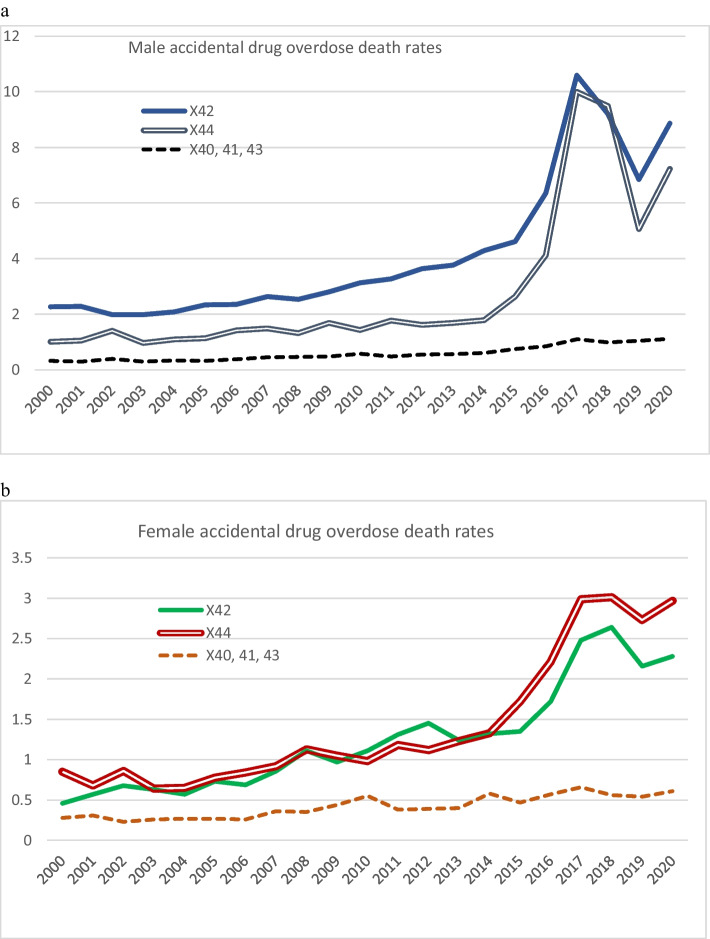
Accidental drug overdose death rates (**a** male, **b** female). Note: ICD-10 codes: X40, accidental poisoning by nonopioid analgesics, antipyretics, antirheumatics; X41, accidental poisoning by antiepileptic, sedative-hypnotic, antiparkinsonism, and/or psychotropic drugs not elsewhere classified; X42, accidental poisoning by narcotics and psychodysleptics (hallucinogens); X43, accidental poisoning by drugs acting on the autonomic nervous system; X44, accidental poisoning by other and unspecified drugs

Figure 2a and b show progressive increases in both opioid (X42) and unspecified (X44) death rates between 2004 and 2017. Among drugs included in the X44 (unspecified/other/polydrug) group are those where coders (persons deciding which ICD-10 code to assign as the c.o.d.) have established that drugs of more than one type may have contributed to the death (for example, fentanyl plus dexamphetamine) and no drug has been specified as primarily responsible for the death. “Opioid-related” deaths include deaths coded X44, where an opioid was taken at the same time as a stimulant and/or a non-opioid drug. Recent evidence cited by the GoC (2022) points to a large proportion of unspecified deaths (X44) being both opioid and stimulant related. The patterns of decrease and then increase of X42 and X44 rates in 2017–2020 (both sexes) were similar to those shown in Fig. 1, whereas there was little change in rates of other drug deaths in 2017–2020. If it is assumed that about 60% of unspecified drug overdose deaths (suicides, accidental, or EUI) involved opioids, calculations based on the data graphed in Fig. 1 suggest that the rate of opioid-related deaths in Canada in 2020 was about 10.0 (male 15.0, female 5.0). Estimates suggest that Canada’s stimulant-related death rate was about 5.0.

Joinpoint analysis showed that progressive increases in accidental opioid overdose death (X42) rates were significant (male 2003–2014 APC 6.6, *p* < 0.001; 2014–2017 APC 31, *p* < 0.001; female 2000–2019 APC 8.0, *p* < 0.001). Male accidental unspecified drug (X44) death rates rose in 2014–2017 (APC 49.7, *p* < 0.05); increases in female rate were not significant.

### Changes in Drug Suicide Rates, 2000–2020

**Fig. 3:**
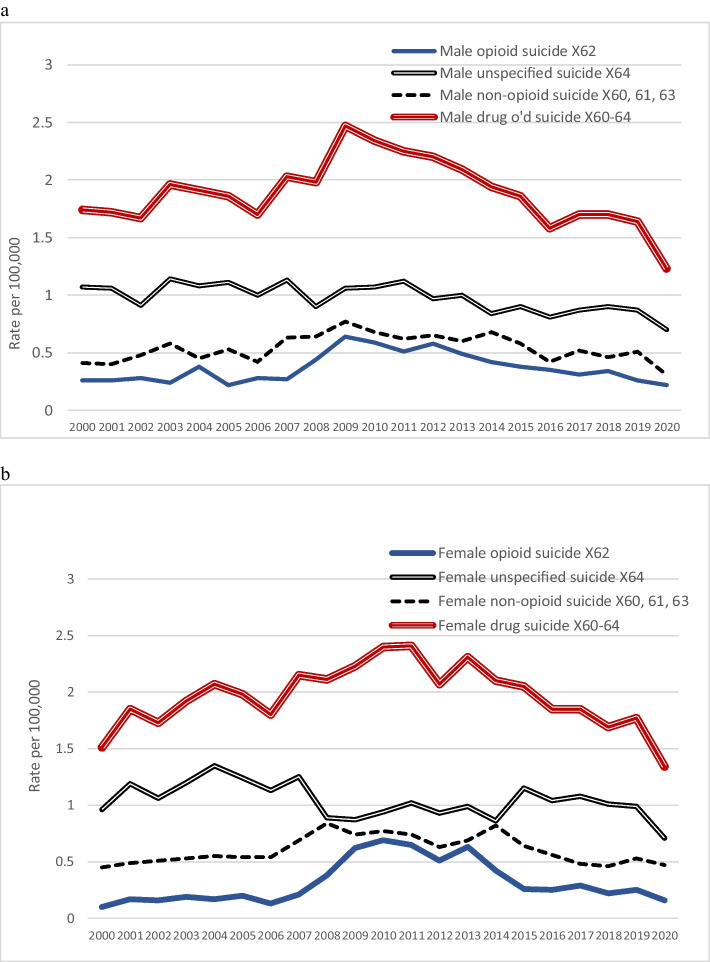
Drug suicide rates (**a** male, **b** female). Note: ICD-10 codes: X60, suicide by taking nonopioid analgesics, antipyretics, antirheumatics; X61, suicide by taking antiepileptic, sedative-hypnotic, antiparkinsonism, and/or psychotropic drugs not elsewhere classified; X62, suicide by taking narcotics, psychodysleptics (hallucinogens); X63, suicide by taking drugs acting on the autonomic nervous system; X64, suicide by taking other or unspecified drugs

Figures 3a and b show that there was an overall increase in drug suicide rates up to 2009–2010, but the male X62, X64, and non-opioid drug suicide rates all fell somewhat from 2011 onwards. The female drug type patterns of rise and fall were less consistent: all rose somewhat in 2000–2008; all were fairly steady between 2008 and 2013. The female X62 and non-opioid drug death rates then progressively fell, but the X64 rate rose during 2014–2016 and then fell. Male rates of EUI opioid overdose death rates were mostly two or three times *higher* than *opioid suicide* rates, except in the early 2000s and in 2016–2020. Apart from male EUI opioid deaths, rates of drug EUI deaths were mostly *lower* than rates of drug suicides, and all (including male EUI opioid death rates) were near zero in 2016–2020.

**Fig. 4:**
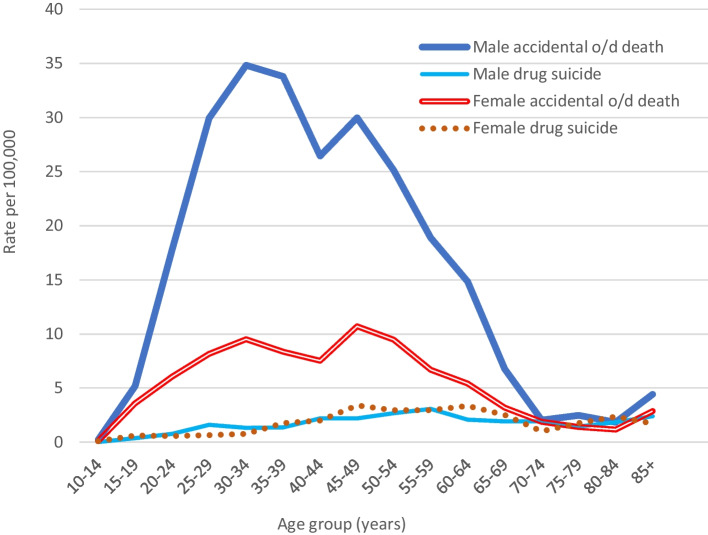
A comparison of age patterns (male and female) of accidental drug overdose deaths (X40–44) and of drug suicides (X60–64), 2014–2018

**Fig. 5:**
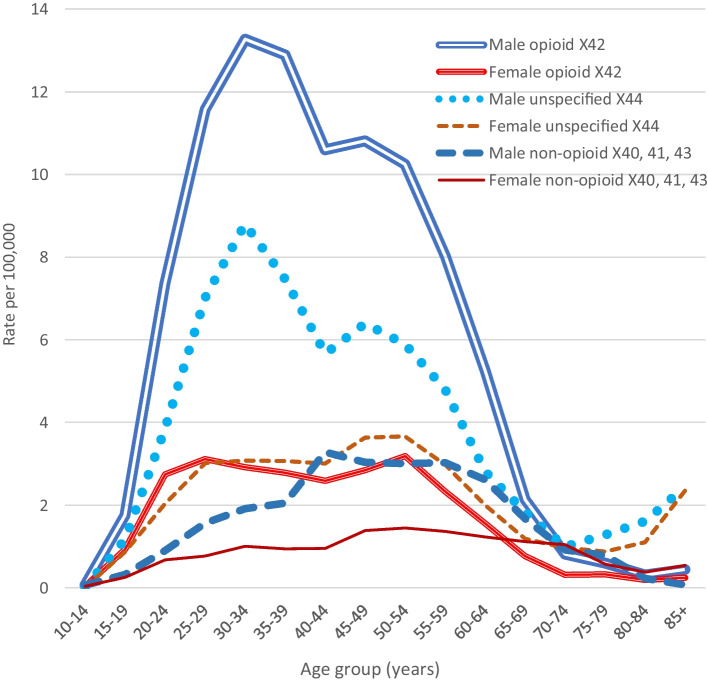
Age patterns of accidental opioid (X42)), non-opioid (X40, 41, 43), and unspecified/other (X44) drug overdose death rates, 2014–2018

The age patterns of male and female accidental drug overdose deaths (X40–44) and drug suicides (X60–64) in 2014–2018 are shown in Fig. 4. The age patterns of male and female drug deaths coded as either X42, X44, or non-opioid are shown in Fig. 5. The male X42 (opioid) rate peaked at age 30–34 years (13.27). The female rate averaged 2.8 between ages 20 and 59 years, but decreased in late life. The patterns of male and female unspecified drug death rates were similar to those of the opioid death rates, but male unspecified rates were lower than opioid rates. Rates of non-opioid drug deaths were relatively low in young adulthood, increasing to a plateau in middle age.

There were significant increases in male rates of EUI drug deaths in 2000–2004 (opioid), 2000–2014 (non-opioid and unspecified), and female rate in 2000–2014 (opioid). There were significant falls in female EUI drug death rates in 2014–2017 (opioid), 2013–2019 (non-opioid), and 2013–2019 (unspecified drug).

The mean annual crude suicide rate in Canada in 2000–2013 was 11.59. In 2014–2018 it was 11.47 (male 17.13, female 5.76). Examination of suicide figures published in 2018 to 2020 shows that “preliminary” suicide figures for those years were *revised upwards* when updated in 2020 to 2022. For example, the male and female 2018 rates first published in 2020 were 15.65 and 4.98, but when revised in 2022 (after coders had taken account of coronial verdicts and added information), they were 18% higher at 18.65 and 5.87, respectively. It will be relevant to examine the extent to which other c.o.d. rates have been revised, one and two years after release of preliminary data. Canada’s mean drug suicide rate rose to 2.07 in 2007–2014, and fell to 1.76 in 2015–2019, and to 1.30 (a “preliminary” figure) in 2020. In 2022, the 2019 drug suicide rate was revised upwards from 1.36 to 1.71. In a majority of age groups, the numbers of female drug suicide deaths per year exceeded the comparable number of male deaths.

Joinpoint analysis showed statistically significant increases in opioid suicide rates in 2006–2010 (APCs male 25.9, female 45.7; *p* < 0.01) and significant decreases in 2010–2019 (APCs male − 10.7, female − 15.0; *p* < 0.001). The decreases were in sharp contrast to the increases in accidental drug death rates across 2011–2019. Similar changes in non-opioid suicide rates were revealed: 2000–2011 APCs male 5.2, female 4.2; and 2011–2019 APCs male − 7.3, female − 11.8 (*p* < 0.001).

### Age Patterns of Drug Suicides and Accidental Drug Deaths

**Fig. 6:**
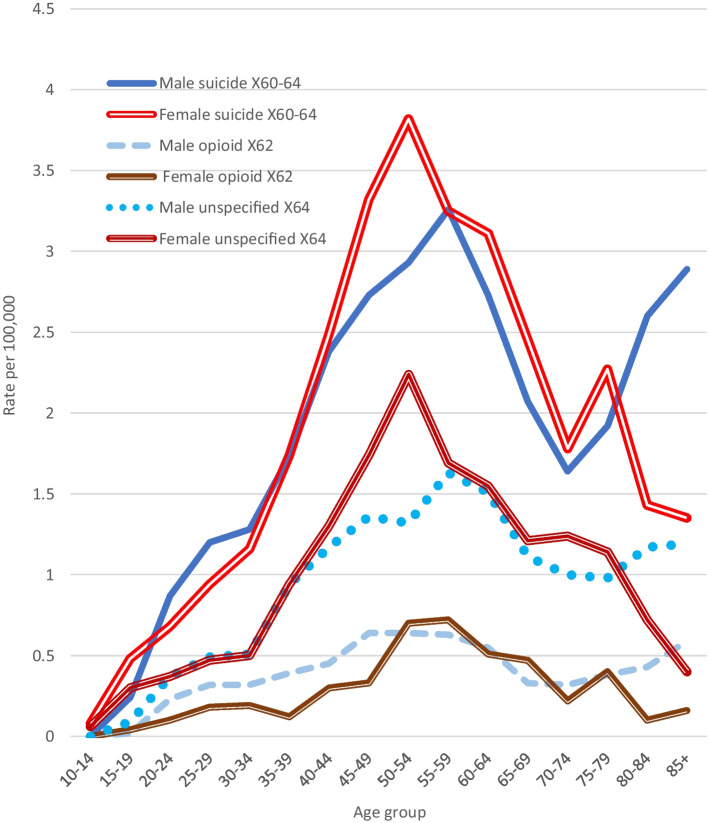
A comparison of age patterns of male and female drug suicide death rates, 2014. Note: ICD-10 codes: X60–64, accidental poisoning by drugs or medications; X62, accidental poisoning by narcotics; X64, accidental poisoning by other and unspecified drugs. For clarity, non-opioid suicide rates (X60, 61, 63) have not been graphed. Patterns of opioid and non-opioid suicide rates were similar, but rates of the latter were higher (peaking at around 50–59 years; male 0.98, female 1.17)

The age patterns of drug suicide in 2014–2018 (Fig. 6) were compared with accidental drug death patterns (Figs. 4 and 5). The male pattern of drug suicide was bimodal, with a first peak at age 55–59 years (3.26) and a second, lower peak in late life (2.89). The female pattern was convex, with a middle age peak of 3.82. The age patterns of unspecified drug suicide deaths were similar to those of opioid suicide deaths except that the rates were nearly three times higher. Peak rates of male and female opioid, unspecified, and non-opioid drug *suicides* were all at age 50–59 years, contrasting with the abovementioned age patterns of accidental drug death rates. Non-opioid accidental and suicide age patterns (both sexes) resembled each other, except that the male non-opioid accidental death rate was higher (Fig. 2a); rates were highest at 40–59 years.

### EUI Deaths

Male and female rates of opioid, non-opioid, and unspecified EUI drug deaths (coded Y10-14) in Canada in 2003–2015 were similar and fairly steady, mean annual rates being, respectively, 0.32, 0.19, and 0.64. In 2016–2019, they were lower, at about 0.07, 0.07, and 0.18 (Fig. 1). The male EUI drug death rate peaked at age 30–39 (rate 2.0), and the female at age 50–59 years (rate 1.36).

### R99 Deaths

**Fig. 7:**
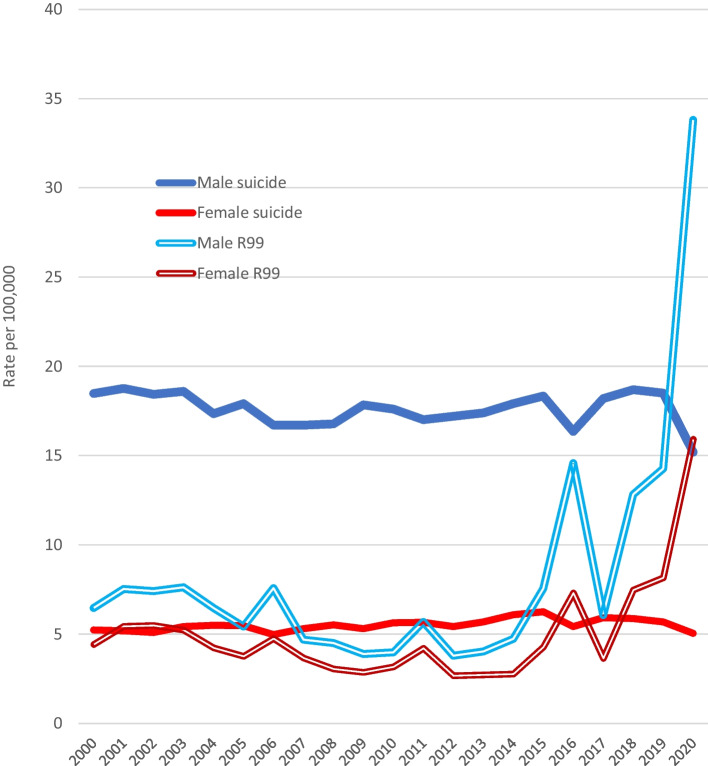
Male and female suicide rates (any method) (X60–84) compared with rates of death due to ill-defined or unspecified cause (R99), 2000–2020

**Fig. 8:**
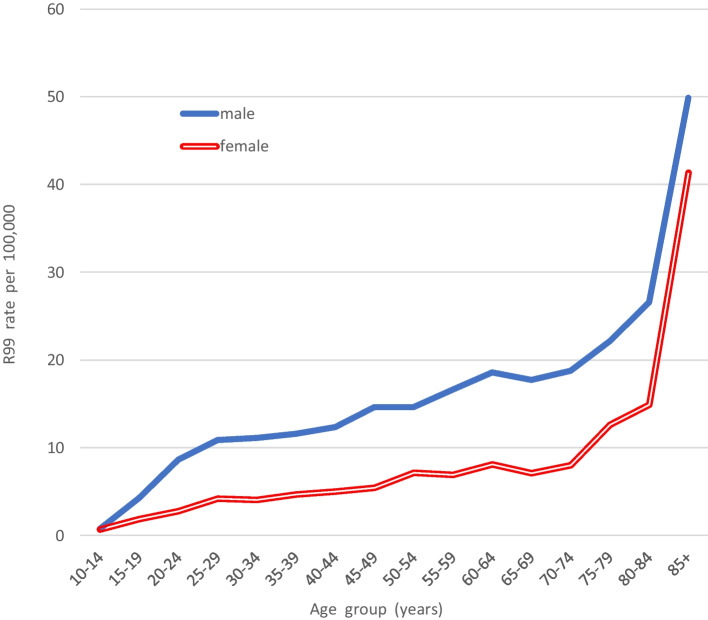
A comparison of age patterns of rates of male and female R99 (ill-defined/unspecified cause) deaths, 2014–2018

The mean annual rates of “ill-defined and unknown cause” deaths coded R99 in 2004–2008 and 2009–2013 were calculated as, respectively, 4.79 and 3.69. In 2014, the rate was 3.73, but it increased progressively over the next few years. In 2022, Statistics Canada reported the 2020 R99 rate was 24.85 (male 33.8, female 15.9). Figure 7 shows male and female R99 rates and suicide rates (any method) during the last two decades, but note that the 2019 and 2020 rates were those being reported in 2022 and are *likely to be revised downwards when updated* in 2023. In 2021, Statistics Canada reported the 2019 R99 rate as 17.78, but in 2022, the “preliminary” 2019 R99 rate was *revised downwards* to 11.23 (male 14.3, female 8.16). As in most nations (Snowdon, 2021a), graphs of the Canadian R99 age patterns in 2014–2018 were upward-sloping (Girard, 1993), rates increasing exponentially in late life to 49.91 (male) and 41.36 (female) at age 85 + years (Fig. 8).

## Discussion

As shown in Fig. 2, there were progressive increases in the Canadian rates of male and female accidental opioid overdose deaths, in the decade up to 2017. Up to 2010, this rise, like in the USA (Ciccarone, 2021), was attributed largely to increased availability of prescription drugs, and pharmaceutical industry promotion in the mid-1990s of opioid drugs such as oxycodone for treatment of chronic pain (Fischer et al., 2021). Around 2010, various intervention and policy measures were introduced in both Canada and the USA, aimed at reducing opioid dispensing, but concurrently, for unclear reasons, the supply of heroin in both countries then increased and its price dropped sharply (De Weerdt, 2019): from 2010 to 2016, deaths from heroin increased fivefold in the USA. From about 2013, heroin dealers began to mix their products with fillers and fentanyl, and a range of cheaper, highly potent illicit/synthetic opioids then proliferated on the drug markets, accelerating opioid-related mortality (De Weerdt, 2019). Our estimate that the rate of opioid-related deaths in Canada in 2017 was about 10.0 is consistent with data provided by the Public Health Agency of Canada (GoC, 2021), but was lower than the corresponding US rate of 14.6 (CDC, 2019).

Also concurrently, but more quickly after 2014, there was an escalating accidental “unspecified” drug death rate. The latter is largely attributable to increased rates of death resulting from use of combinations of opioids and stimulants, coded as unspecified (X44) because more than one type of drug was viewed as contributory (Fig. 2). There was a similar increase in stimulant use in the USA, where the rate of deaths due wholly or partly to psychostimulant use quadrupled between 2013 and 2019, from 1.2 to 5.0, one-third being in combination with synthetic opioids (Mattson et al., 2021). In 2017, two-thirds of US drug deaths involved an opioid, 19.8% involved cocaine, and 14.7% amphetamines or other psychostimulants (Kariisa et al., 2019).

The fall in drug-related deaths in 2018–2019 in both the USA (Ciccarone, 2021) and Canada was thought to be driven by declines in deaths related to prescription opioids. However, from mid-2019, there was a resumed increase in synthetic opioid overdose in Canada (Fig. 2). The COVID-19 pandemic contributed to the already deadly and ongoing national public health overdose crisis, and the rate of apparent opioid toxicity deaths in 2021 was 20.0 (GoC, 2022). As mentioned, 59% of these deaths involved a stimulant. Of the apparent stimulant toxicity deaths in 2021, 86% involved an opioid, while 62% involved cocaine and 55% methamphetamines. In the USA, the opioid-related death rate rose to 21.6 in 2020 (CDC, 2022), predominantly attributable to fentanyl and its analogues (Ciccarone, 2021). Referring to increasing co-use of stimulants and opioids in North America, Ciccarone (2021) commented that “although opioids have dominated the ‘triple wave epidemic’ (opioids, heroin, and then fentanyl) of drug-related overdose deaths, a ‘fourth wave’ of high mortality involving cocaine and methamphetamine use has been gathering force”, a view echoed by Fischer et al. (2021) when calling it a “twin” epidemic.

Surprisingly little has been written about drug suicides in Canada during recent years even though rates of accidental overdose deaths have caused considerable concern. The present study demonstrates that Canadian opioid and non-opioid suicide rates rose as use of prescribed opioids increased around 2006–2012, though without a rise in unspecified drug suicides. Opioid and non-opioid suicide rates reportedly halved between 2014 and 2019, while accidental opioid and unspecified drug rates increased several-fold, and the non-opioid drug death rate doubled. In relation to the last decade, our hypothesis that increasing accidental drug overdose rates would be found to be associated with increased drug suicide rates was thus refuted.

The differing age patterns shown in Figs. 4 and 5 appear to confirm the opinion of Chan et al. (2018) that accidental and suicide opioid-toxicity deaths occur in distinct populations, but suggest that the same applies to unspecified accidental drug (mostly stimulant or polydrug) deaths versus drug suicides, whereas age patterns of accidental and suicide non-opioid deaths are more similar.

A question needs to be posed. The opioid crisis and the more recent stimulant crisis are realities, but is it possible that changes in policies or procedures in Canada have led to misclassification of some drug suicides during the last few years? For years, Rockett et al., (2015, 2021) have commented that drug-intoxication suicides are highly prone to be misclassified as accident or undetermined. Yet it has been asserted that, in recent years, little attention has been given, in the USA, to the contribution of suicide to overdose mortality (Oquendo & Volkow, 2018) and there has been inadequate screening for suicide risk. Rockett et al. (2015) suggested that identification of suicide as such is often obfuscated by death investigations that are inadequate for validly differentiating manner of death. “The lack of adequate field investigations – including standardized in-depth data collection procedures or psychological autopsies, which can improve the sensitivity of death investigations, further complicates accurate manner of death determination for drug-caused suicides” (Rockett & Caine, 2020). The increase in drug overdose deaths in 2006–2010 was largely attributed to over-prescribing of drugs by doctors. The fact that drug suicide rates reportedly increased almost as much as accidental drug overdose death rates over that time is likely to have been because doctors could have observed (and later reported to investigators about) the mental status of people to whom they prescribed drugs. Over the next decade, when opioids and other drugs were becoming more readily available on the streets and were increasingly obtained without seeing doctors to obtain prescriptions, documented drug suicide rates progressively decreased (Fig. 3). Substance use disorders are recognized as strong determinants of suicide, but it is also found that they diminish the likelihood of a suicide ruling (Rockett et al., 2010). Stone et al. (2017) summarized opinions of US experts convened to discuss classification barriers in relation to determining manner of death in “drug intoxication deaths.” They noted system-level factors that contribute to inconsistencies between jurisdictions—in definitions, practices, training, beliefs, observer bias, toxicological testing, resourcing, and more. Across the USA, there are substantial differences in the percentages of drug deaths that are classified as “undetermined” (Choi et al., 2019; Warner et al., 2013). There are comparable inconsistencies between Canadian jurisdictions.

Suicide rates officially reported by most countries are widely believed to be lower than actual rates, with varying proportions of suicides “hidden” as accidents, EUI deaths, or having ill-defined or unknown cause (Snowdon & Choi, 2020). Rockett et al. (2015) regard EUI deaths as “the manner-of-death category most prone to contain misclassified suicides”. Snowdon (2021a) compared rates of suicide and of deaths coded to EUI, accidental poisoning and R99 in 2015 in various countries that provide high-quality mortality data. In Canada, the EUI rate (poisoning and all other injuries) in 2013 was 1.87. Poisoning has been the method most often recorded (64%) in Canadian cases of EUI (Lachaud et al., 2018). The EUI rate in Spain in 2015 was 0.10, but in most other nations, it was above 1.0 (Snowdon, 2021b). The Canadian EUI rate fell sharply in 2016 and averaged 0.32 in 2016–2020 (Fig. 1); thus, recent decreases in Canada’s suicide rate cannot be attributed to concurrent changes in EUI rates, and our hypothesis that the drug EUI rate would have been found to increase along with the drug overdose death rate was refuted. The fact that the Canadian drug suicide rate decreased in 2015 to levels about the same as before 2007 (Fig. 3) raises two questions: Was this a real fall in suicide rate? Is the decrease attributable to suicides being “hidden”? The possibility of their being “hidden” as accidents could only be addressed by rigorous investigation of a series of overdose deaths, including psychological autopsy. Even then, in the absence of evidence about intent to die, it could be expected that the EUI rate would have escalated rather than fallen.

Another possibility is that changes in procedures or policies could have led to some (or many) suicides being misclassified as due to ill-defined or unknown cause (R99). The R99 rate in Canada, having been fairly steady at about 4.0 for a decade, increased considerably during 2017–2020 (Statistics Canada, 2022). Rockett et al. (2011) stated that poisoning suicides are highly prone to misclassification by officials as (a) injury of undetermined intent or (b) ill-defined and unknown cause. Bakst et al. (2016) found the category of “unknown causes” to be a prime contender for containing misclassified suicides, even though the percentage of probable suicides among R99 cases was under 2%. In general, graphs of age patterns of R99 rates in different nations show increases that become exponential in late life, and Spain provides a classic example (Snowdon, 2021b). Canada’s R99 rates in 2014–2018 showed a pattern similar to Spain’s, the male rates being twice as high as the female up to late old age (Fig. 7). The fact that R99 rates progressively increased more than fourfold between 2017 and 2020, at a time when drug suicide and EUI rates were progressively falling (Fig. 8), raises the possibility that an increasing number of suicides in recent years were being misclassified as R99 deaths. It is known that policies in some jurisdictions allow coders to assign an R99 code as a “temporary” measure when decisions about c.o.d. are “pending.” It is also known that illicit drug users are disinclined to admit that an overdose was intentional even if, in a fluctuating way, it was. Again, this possibility can only be researched by ensuring rigorous investigation (including toxicology) in a large series of deaths where the possibility of suicide cannot be excluded. Such investigations could be expensive and the “yield” small, which may explain why Bakst et al.’s study (2016) remains relatively unique.

### Limitations

The numbers of opioid-related deaths reported in various recent publications sometimes differ from those calculated from data provided by Statistics Canada. Such differences may be because sources (such as the Public Health Agency of Canada) obtain c.o.d. figures directly from provincial and territorial coroners and medical examiners, rather than relying on data coded by Statistics Canada. ICD-10 codes cocaine deaths among opioid/narcotic deaths rather than as a psychostimulant death, and this applies in Figs. 2–5. Differences do not necessarily matter; changes in rates and numbers may be of more importance than the figures themselves. Differences also could arise by different interpretations concerning which of a combination of drugs should be recorded as the prime cause of a drug death.

When this paper was first drafted, Statistics Canada told us (personal communication, July 27, 2021) that mortality data provided in their on-line database for the years 2017 and 2018 had not been updated since their publication as “preliminary” data, and results of their historical revision of microdata for the years 1991–2017 had not yet been released. During the year since then, Statistics Canada has published its historical revision and revised more recent data. All data that were first published more than two years previously can be considered finalized. Studies from other countries have shown that, when mortality data are initially published, they often (especially when released within two years of death) show high R99 rates; these R99 numbers commonly are revised downwards after one or two years, as authorized officers finalize verdicts in cases that were still open when preliminary mortality data were published. As a corollary, numbers and rates of suicide, accidental death, and EUI could all mount as the R99 numbers come down. We commend Canada for now ensuring that revisions to preliminary data are made annually for at least two years after first release of those data. Nevertheless, there remain reasons for believing that sizeable percentages of drug suicides in Canada are being misclassified as accidental or as deaths of unknown cause.

## Conclusion

This study provides continuing reasons for concern about escalating rates of opioid and stimulant deaths. There was a temporary downturn in 2017–2019, but the COVID pandemic appears to have ushered in even greater cause for alarm about mortality rates. Unexpectedly, and contrary to our hypotheses, recent data show falls in drug suicide and drug EUI rates, coinciding with increases in accidental overdose deaths, and demonstrate a co-occurring rise in deaths coded as due to ill-defined or unknown cause. This is despite recognition of enhanced suicide risks among those diagnosed with substance use disorders, and those who take excessive drugs to relieve mental or physical pain. It is plausible that suicides are not being identified as such, partly because the possibility of suicide is inadequately considered and relevant inquiries are not conducted. For that reason, it is recommended that there should be increased resourcing for interviews with close informants, allowing investigation regarding thoughts, plans, and attitudes of those who die from drug overdoses.

## Data Availability

All data documented in this paper are in the public domain, as cited in References.
